# Moderate Frequency Resistance and Balance Training Do Not Improve Freezing of Gait in Parkinson's Disease: A Pilot Study

**DOI:** 10.3389/fneur.2018.01084

**Published:** 2018-12-10

**Authors:** Christian Schlenstedt, Steffen Paschen, Jana Seuthe, Jan Raethjen, Daniela Berg, Walter Maetzler, Günther Deuschl

**Affiliations:** Department of Neurology, University Hospital Schleswig-Holstein, Christian-Albrechts-University, Kiel, Germany

**Keywords:** freezing, postural control, balance, Parkinson's disease, exercise, training

## Abstract

**Background and Aim:** Individuals with Parkinson's disease (PD) and Freezing of Gait (FOG) have impaired postural control, which relate to the severity of FOG. The aim of this study was to analyze whether a moderate frequency resistance (RT) and balance training (BT), respectively, are effective to diminish FOG.

**Methods:** This *post-hoc* sub-analysis of a randomized controlled training intervention study of PD patients with and without FOG reports about results from FOG patients. Twelve FOG patients performed RT and 8 BT (training 2x/week, 7 weeks). Testing was performed prior and post intervention. FOG was assessed with the FOG Questionnaire (FOGQ) and with the FOG score of a FOG provoking walking course. Balance performance was evaluated with the Fullerton Advanced Balance (FAB) scale. Tests were conducted by raters blinded to group allocation and assessment time point (only FOG score and FAB scale).

**Results:** For the FOGQ and FOG score, no significant differences were found within and between the two training groups (*p* > 0.05) and effect sizes for the improvements were small (*r* < 0.1). Groups did not significantly improve in the FAB scale. FOG score changes and FAB scale changes within the RT group showed a trend toward significant negative correlation (Rho = −0.553, *p* = 0.098).

**Conclusions:** Moderate frequency RT and BT was not effective in reducing FOG in this pilot study. The trend toward negative correlation between changes in FOG score and FAB scale suggests an interaction between balance (improvement) and FOG (improvement). Future studies should include larger samples and high frequency interventions to investigate the role of training balance performance to reduce the severity of FOG.

## Introduction

Freezing of gait (FOG) in Parkinson's disease (PD) is a disabling symptom which is defined as the “brief, episodic absence or marked reduction of forward progression of the feet despite the intention to walk” (1). It has been shown that FOG-specific training interventions, such as cueing, can reduce FOG (2–4). It is however unclear whether non-FOG-specific exercises which target FOG-related deficits also alleviate FOG.

Individuals with PD with FOG (PD + FOG) have postural control deficits (5, 6) and the severity of FOG relates to the degree of postural instability (5). Recently it has been shown that PD+FOG have smaller anticipatory postural adjustments (APAs) when preparing for step initiation compared to patients without FOG (PD-FOG) and that the size of medio-lateral APAs was positively correlated with FOG severity (7). It has been suggested that reducing the size of APA might be a compensatory strategy addressing postural control deficits (7). Further, Plotnik et al. (8) proposed that FOG might be a result of multiple with FOG associated motor impairments such as dynamic postural control, gait asymmetry, and gait variability. According to this framework, FOG might occur if enough of these features deteriorate. It is unclear, whether an improvement of postural control as one of these FOG related features might diminish FOG.

In a recent study we compared resistance training (RT) with balance training (BT) to improve postural control in PD and we showed that RT was beneficial to improve balance performance. This sub-analysis has two aims: first, to test whether RT or BT is effective to reduce the severity of FOG and second, whether an improvement in FOG is related to improved postural control.

## Materials and Methods

This study is a sub-analysis (only PD + FOG, *N* = 20) of a randomized controlled trial that investigated the efficacy of RT vs. BT to improve postural control in PD (*N* = 40) (9).

### Participants

Inclusion criteria were the diagnosis of idiopathic PD as defined by the UK Brain Bank criteria, FOG based on the FOG Questionnaire (10) (FOGQ) (item 3 > 0) and postural instability [Fullerton Advanced Balance (FAB) scale <26 points (11)]. Details about the exclusion criteria are reported in Schlenstedt et al. (9). Individuals had to be on stable medication during the training and assessment periods.

This study was carried out in accordance with the recommendations of Ethik-Kommission, Universitätsklinikum Schleswig-Holstein, Campus Kiel, Arnold-Heller-Straße 3, 24105 Kiel, Germany, with written informed consent from all subjects. All subjects gave written informed consent in accordance with the Declaration of Helsinki. The protocol was approved by the Ethik-Kommission, Universitätsklinikum Schleswig-Holstein, Campus Kiel, Arnold-Heller-Straße 3, 24105 Kiel, Germany.

### Randomization and Intervention

Participants were randomized into either RT or BT (7 weeks, 2x/week, 60 min per session) within the original study. There was no stratification for FOG in the original randomization. Training was conducted in groups of 4–5 people. Each session started with a warm-up (10 min) followed by either RT or BT. In brief, RT consisted of lower limb muscle strength exercises and participants' own weight, cuff weights, and elasticated bands were used as resistance. Squats, knee extensions, toe/calf raises, hip abductions, and other exercises were performed [for details see (9)].

BT consisted of static and dynamic postural control tasks. Participants were asked to train their limits of stability by leaning forward/backward/sideward. Reactive postural control was trained by shoulder pulls. One option to reach training progression was the inclusion of unstable surfaces on which the participants had to stand or walk [see Schlenstedt et al. (9) for further details about training progression].

### Testing Procedure and Outcome Measures

Participants were tested 1 week prior (PRE) and 1 week post (POST) intervention. Testing was conducted in the ON state of medication at the same time of a day for each participant. Severity of FOG was assessed with the FOGQ (10) and with the FOG score by Ziegler et al. (12). The FOGQ was conducted by an assessor blinded to group allocation. Trials of the FOG score were video-recorded and videos were rated by an independent rater, also blinded to assessment time point and group allocation.

Furthermore, the following tests were included in the analysis: FAB scale (to assess postural control) (11, 13), Unified Parkinson's Disease Rating Scale (UPDRS), and Mini Mental State Examination.

### Statistical Analysis

Demographic and baseline differences between groups were analyzed with a Mann-Whitney-*U*-Test (except for gender: Chi-Square Test). As data were not normally distributed, non-parametric tests were used. A Wilcoxon-Signed-Rank-Test was conducted to analyze the changes from PRE to POST within one group. To compare the different training types, the differences from PRE to POST were calculated and the magnitude of change were compared between the two groups were analyzed with using the Mann-Whitney-*U*-Test. Effect sizes were calculated (*r* = *z*-score/(n)^∧^1/2). We considered effect sizes to be small with 0.1 < *r* < 0.3, medium with 0.3 < *r* < 0.5 and large with *r* > 0.5 (14). The magnitude of change in FOG severity was correlated [Spearman's rank correlation coefficient [Rho] with the change in balance performance (FAB scale). Level of significance was set at *p* < 0.05. Statistical analysis was performed with R (version 1.1.442) (15).

## Results

**Figure 1:**
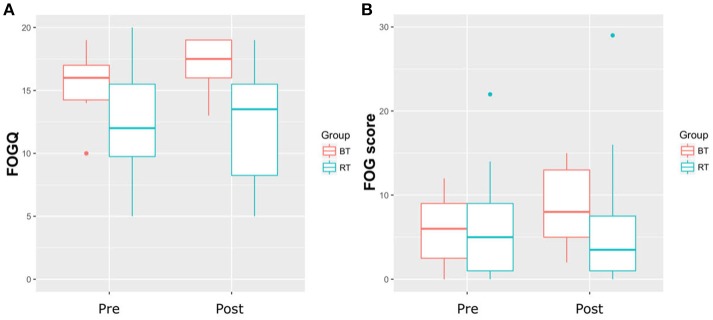
**(A)** Results of the Freezing of Gait Questionnaire (FOGQ). **(B)** Results of the Freezing of Gait score; BT, Balance Training; RT, Resistance Training.

Table 1 shows the participant characteristics. RT and BT groups neither significantly differed in any demographic variable, nor with regard to severity of FOG. Both training types had no significant effect on FOGQ and FOG score (Table 2 and Figure 1) (*p* < 0.05). The effect sizes for the slight improvements within the RT group were small (*r* < *0.1*) (14). Within this sample, the groups did not improve significantly in postural control as measured with the FAB scale (*p* < 0.05). Although statistically not significant within this sample, a large effect was found within the RT group when relating the change in balance performance (FAB scale) with the change in FOG score (Rho = −0.553, *p* = 0.098). A similar trend was found when calculating this correlation taken both groups together (*p* = 0.11, Rho = −0.4). A medium effect was found within the BT group (*p* = 0.426, Rho = 0.361). Changes in FOGQ was not related to the change in FAB scale (RT: *p* = 0.948; BT: *p* = 0.612). The exclusion of outliers did not relevantly affect our results.

**Table 1:** Participant characteristics.

**Variable**	**Resistance training (*n* = 12)**	**Balance training (*n* = 8)**	*p*-value^*^
Age (y)	78.3 (5.8)	81.4 (7.3)	0.41
Gender (M/F)	9/3	6/2	1.00
BMI (kg/m2)	27.3 (6.5)	24.5 (3.5)	0.34
Disease duration (y)	11.2 (6.6)	8.4 (7.3)	0.38
HandY stage	2.8 (0.3)	2.9 (0.5)	1.00
UPDRS	43.2 (13.2)	45.8 (9.8)	0.51
UPDRS III	24.3 (10.0)	25.8 (5.7)	0.61
FAB scale	21.1 (4.7)	22.4 (5.2)	0.61
MMSE	27.4 (3.7)	26.2 (4.0)	0.44
LEDD	765 (448)	652 (286)	0.46
FOGQ	12.5 (4.5)	15.3 (3.1)	0.22
FOG score	6.6 (7.2)	5.9 (4.4)	0.92

*Values represent mean (SD) or number*.

* *p-value of Mann-Whitney-U-Test (and Chi-Square-Test for Gender)*.

**Table 2:** Statistical results of the FOGQ and FOG score.

**Test**	**Group**	**Value**	**PRE**	**POST**	**Within group comparison from PRE to POST**		**Between group comparison of changes from PRE to POST**	

					***p***-value^*^	**Effect size r**	***p***-value^**^	**Effect size** ***r***
FOGQ	RT	Mean (SD)	12.5 (4.5)	12.3 (4.8)	0.878	0.031	0.279	0.255
		Median (Range)	12 (5–20)	13.5 (5–19)				
	BT	Mean (SD)	15.3 (3.1)	17.0 (2.4)	0.136	0.430		
		Median (Range)	16 (10–19)	17.5 (13–19)				
FOG score	RT	Mean (SD)	6.6 (7.2)	6.9 (9.1)	0.833	0.047	0.153	0.347
		Median (Range)	5 (0–22)	3.5 (0–29)				
	BT	Mean (SD)	5.9 (4.4)	8.7 (5.1)	0.105	0.433		
		Median (Range)	6 (0–12)	8 (2–15)				
FAB scale	RT	Mean (SD)	21.1 (4.7)	23.2 (5.0)	0.245	0.336	0.534	0.139
		Median (Range)	22 (10–29)	22.5 (15–34)				
	BT	Mean (SD)	22.4 (5.2)	22.4 (5.7)	1.000	0.000		
		Median (Range)	23.5 (15–27)	25.5 (12–27)				

* *p-value of Wilcoxon Signed Rank Test*.

** *p-value of Mann-Whitney-U Test. RT, Resistance Training; BT, Balance Training*.

## Discussion

We could not show that a moderate frequency RT and BT is effective to diminish FOG in people with PD in the present pilot study. As FOG-specific training interventions such as cueing did indeed show statistically significant reduction in FOG severity (2–4), our study might indirectly supports the hypothesis that exercises specifically designed to target FOG might be more beneficial than non-FOG-specific interventions. We acknowledge that our sample was small and results have to be interpreted cautiously; however, due to the low effect sizes we do not expect reaching significant results with this training protocol even with a larger sample. We rather believe that increasing the intensity and frequency of training is required to see a relevant effect and this has been suggested by other larger trials (16).

As FOG is related to postural control deficits (5, 6) the idea of this project was that improved postural control might lead to a reduction in FOG episodes. In the original study, participants of the RT group significantly improved postural control whereas the group of BT did not. We found a large effect when correlating the change in balance performance with the change in FOG severity within the RT group, indicating that those participants who improved postural control may also benefit with respect to FOG, supporting our hypothesis with respect to study aim II. However, this failed to reach statistical significance within this sample and the subgroup of participants with FOG did not significantly improve their postural control in this sub-analysis. This might be explained by the low training frequency (2x/week) and by the small subsample size, as in the original study on all participants of the RT balance performance improved significantly (9). Thus, the impact of training balance performance on FOG cannot clearly be answered with this study.

The following limitations have to be mentioned: Sample size is small and results therefore have to be interpreted cautiously. This study did not include a non-exercise control group which would give additional information with respect to the training effects.

A moderate frequency RT and BT was not effective to diminish FOG within this small sample. This pilot study might help designing future studies which should include larger samples and higher training frequency to investigate the role of training balance performance to reduce FOG occurrence in PD.

## Author Contributions

CS: Study design, study conduction, data collection, statistical analysis, and writing of first draft; SP and JS: Data analysis and manuscript critical revision; JR and GD: Study design and manuscript critical revision; DB and WM: Manuscript critical revision.

### Conflict of Interest Statement

The authors declare that the research was conducted in the absence of any commercial or financial relationships that could be construed as a potential conflict of interest.
